# Assessment of global asymptomatic SARS-CoV-2 infection and management practices from China

**DOI:** 10.7150/ijbs.59374

**Published:** 2021-03-10

**Authors:** Zheng Chen, Bili Wang, Shanshan Mao, Qing Ye

**Affiliations:** The Children's Hospital, Zhejiang University School of Medicine, National Clinical Research Center for Child Health, National Children's Regional Medical Center, Hangzhou 310052, China.

**Keywords:** SARS-CoV-2, asymptomatic infection, COVID-19

## Abstract

With ongoing research, it was found that asymptomatic severe acute respiratory syndrome coronavirus 2 (SARS-CoV-2) infection was widespread in coronavirus disease 2019 (COVID-19) populations. Studies have confirmed asymptomatic patients with COVID-19 have potential infectivity, and most of the transmission occurred before symptoms appear. Asymptomatic infection rates varied widely in different countries and regions. Identifying the asymptomatic infected persons and cutting off the infection source is an effective way to prevent the spread of this disease. However, asymptomatic patients have hidden clinical symptoms, and screening based only on the clinical symptoms of COVID-19 can easily lead to a missed diagnosis. Therefore, determining asymptomatic infection patients by SARS-CoV-2 nucleic acid testing is the gold standard. A series of prevention and control measures adopted by the Chinese government, especially the “Four Early” policy, have achieved outstanding achievements, which are worth learning from by other countries.

## Introduction

Coronavirus disease 2019 (COVID-19), the disease caused by severe acute respiratory syndrome coronavirus 2 (SARS-CoV-2), is a contagious disease that can cause severe respiratory diseases 1. It can also induce inflammatory factor storms in some infected people, causing acute respiratory distress syndrome (ARDS) and multiple organ dysfunction or even death 2-9. At present, COVID-19 is spreading rapidly around the world 10, which is a global public health emergency that urgently needs to be resolved 11. With the development of the epidemic, many studies have shown that of people infected with SARS-CoV-2, a certain proportion is asymptomatic. Asymptomatic infection refers to the positive detection of SARS-CoV-2 nucleic acid in patient samples through reverse transcription-polymerase chain reaction (RT-PCR) though the patients do not have symptoms 12. Increasing evidences show that many COVID-19 patients have no symptoms but can spread the virus to others. Management of and risk assessment for asymptomatic infections have also become one of the main difficulties faced by the current epidemic prevention and control measures. This article analyzes the definition of asymptomatic infection and morbidity, infectivity, prevention and treatment, hoping to provide some reference for global epidemic prevention and control.

## The definition and characteristic of asymptomatic SARS-CoV-2 infection

Asymptomatic SARS-CoV-2 infections refer to those with no relevant clinical symptoms, such as fever or cough, but whose respiratory tract specimens or stool specimens or urine specimens et al. were test positive for SARS-CoV-2. Asymptomatic infections are mainly found by screening in four aspects: one is the cluster epidemic investigation; the second is the tracking investigation of the infection source; the third is close contacts testing; and the fourth is the large-scale nucleic acid screening. Asymptomatic SARS-CoV-2 infections are mainly divided into two categories 12: ① The infected person has a positive SARS-CoV-2 nucleic acid test. However, after the 14-day incubation period, there are no clinical symptoms or signs; ② RT-PCR is positive for the infected person, though there are no clinical symptoms or signs at the time of sampling, but later, there are clinical manifestations related to COVID-19, namely, the “asymptomatic infection” in the incubation period (incubation period: the period from the pathogen invades the body to the earliest clinical symptoms or signs appear).

Studies have shown that at a skilled nursing facility in Washington, 56% of SARS-CoV-2 positive patients were asymptomatic at the time of testing, and 88.9% of them subsequently developed COVID-19 clinical symptoms 13. In Nanjing, China, none of 24 asymptomatic patients showed obvious symptoms during nucleic acid screening. But then, 5 (20.8%) developed fever, cough, fatigue and other typical clinical symptoms of COVID-19. However, 7 cases (29.2%) showed asymptomatic and normal CT images during whole hospitalization 14. Further research found that those with asymptomatic infections were mostly young patients (<15 years old) and they were also less likely to develop severe pneumonia later 15. However, some studies have found that some asymptomatic patients may be infected for as long as three weeks, and those infected may also develop severe disease 16. These results indicate that asymptomatic SARS-CoV-2 infection is widespread in COVID-19 populations.

## Asymptomatic patients with COVID-19 had potential infectivity and most of the transmission occurred before symptoms appear

Are asymptomatic SARS-CoV-2 infections contagious? Previous studies have reported that surviving SARS-CoV-2 was isolated from asymptomatic patients 17 and that one asymptomatic patient may cause five infections 18. According to results on short-term continuous infection with SARS-CoV-2 and the shedding of the virus, it can be speculated that most of the transmission occurred before symptoms appear 19, 20. Additionally, the viral load detected in some asymptomatic patients was similar to that detected in symptomatic patients 21. All of the above evidence suggests that asymptomatic patients have the potential to transmit the virus. It is necessary to understand the incubation period of SARS-CoV-2 to control the spread of SARS-CoV-2 by asymptomatic patients. As is known to us all, when the serial interval of COVID-19 is shorter than the incubation period, the presymptomatic transmission will occur more frequently than transmission after symptoms appear. Some studies have estimated that the median serial interval of COVID-19 was 2.6-4.0 days, close to or less than the median incubation period of 5 days 22-24. Comparing the case interval time between COVID-19 and SARS (8.4 d), the case interval time of COVID-19 is shorter than that of SARS, which also explains why COVID-19 is significantly more infectious than SARS 25. These studies suggest that it is very challenging to contain the spread of SARS-CoV-2 only by isolating confirmed patients with symptoms 25, 26. There is a need to strengthen the management of asymptomatic infections.

## Asymptomatic infection rates varied widely in different countries and regions

Statistics on the incidence of asymptomatic infections can help clarify the epidemiological potential of COVID-19 transmission and understand the true universality of the disease. At present, the epidemic has spread to more than 200 countries around the world, especially America and Europe. Ascertaining the prevalence of COVID-19 in each country, especially the asymptomatic infection rate, is extremely important for preventing and controlling the world epidemic. Asymptomatic infection rates vary widely in different countries and regions, from 1.2% to 74.8% (**Table 1**). In Europe, Spain has been one of the countries hardest hit by the COVID-19 outbreak. The results of a national SARS-CoV-2 screening study for Spain showed that the positive rate of SARS-CoV-2 was approximately 5.0% (3053/61075), with asymptomatic patients accounting for 21.9% to 35.8% 27. A study collected blood samples from 16,025 people in ten regions of the United States and found that SARS-CoV-2 positive rates ranged from 1.0% to 6.9%, and up to 40.0% of cases were considered asymptomatic 28. In Belgian long-term care facilities, 280,427 people underwent RT-PCR. A total of 8,343 people (3.0%) tested positive and no symptoms were reported for 6,244 (74.8%) of 8,343 people who tested positive 29. In Iceland, 100 (0.8%) positive results were reported among 13,080 SARS-CoV-2 nucleic acid test participants, and 43 (43.0%) were asymptomatic at the time of the test 30. Breslin et al. 31 studied 43 pregnant women infected with SARS-CoV-2 in New York City hospitals and found that 14 (32.6%) had no clinical symptoms. From above, in Europe and America, the proportion of asymptomatic infections patients accounted for 21.9%-74.8% of SARS-CoV-2 infection patients. However, according to the CDC analysis of 72,314 COVID-19 cases in China, there were 889 asymptomatic cases, accounting for only 1.2% of the total number of cases 32. This may be because some studies do not strictly exclude patients in the incubation period. In addition, China's low asymptomatic infection rate may be due to its strict control measures, including wearing masks and avoiding crowds.

SARS-CoV-2 infection rate and the proportion of asymptomatic patients are also different in different regions of the same country. In Spain, in high-risk areas such as Madrid, the COVID-19 incidence rate was more than five times higher than in most coastal provinces. The urban positive rate for areas with more than 100,000 residents was 6.4%, while the positive rate for areas with less than 5000 residents was only 2.0% 27. This suggests that the denser the population, the higher the SARS-CoV-2 positive rate is.

Social distancing is another important factor affecting the spread of SARS-CoV-2. At a skilled nursing facility in Seattle, Washington, 57 (64.0%) of 89 residents tested positive for SARS-COV-2, and three residents showed no symptoms 13. However, in another senior independent and assisted living community in Seattle, 6 of the 142 residents and staff tested were positive for SARS-CoV-2, and the detection rate was 4.2% 33. The small number of COVID-19 cases in this residential community may have increased social distance between residents and decreased contact with healthcare providers. In addition, strict quarantine and protective measures were implemented as soon as possible after two COVID-19 cases were confirmed, thus effectively reducing the spread of the virus. Similarly, another study investigated 565 Japanese citizens evacuated from Wuhan, 8 people (1.4%) were diagnosed with SARS-CoV-2 infection. Among them four people (50.0%) were asymptomatic 34. Similar to the results of the study, among the 3,711 passengers and crew of the Diamond Princess, a total of 634 (17.1%) tested positive, of which 328 (51.7%) were asymptomatic 35. Therefore, we hypothesized that social proximity not only promotes the spread of SARS-CoV-2, but also increases the proportion of asymptomatic infections.

A total of 2147 close contacts were tracked and investigated. The results showed that the total infection rate was 6.2%, and the asymptomatic infection rate accounted for 16.7% of the total number of cases 36. In Taiwan, among 2761 close contacts, there were 22 secondary cases (0.8%) of COVID-19, and 4 cases (18.2%) were asymptomatic infections 21. These suggest that proper screening of close contacts is also crucial to determine asymptomatic infection rates accurately.

The above research results show that there are asymptomatic patients with COVID-19 in different countries and regions, and the proportions are uneven. Controlling population density and maintaining social distance are effective ways to control infection. Screening of close contacts is an effective way to find asymptomatic infection persons.

## The Chinese government's experience in the management of asymptomatic patients with COVID-19

Asymptomatic infection is contagious, and there is a risk of transmission. The Chinese government implements normalized epidemic prevention and control, based on the SARS-CoV-2 nucleic acid test and the SARS-CoV-2 antibody test, and implements the “four early” policy (early detection, early reporting, early isolation, and early treatment) to achieve precise control and rapid treatment of the epidemic. It has accumulated a wealth of practical experience in managing and controlling asymptomatic infections and achieved remarkable effect. The specific measures are as follows:

The first is to improve the prevention and treatment plan. Step up to take a certain percentage of samples in key areas of the epidemic; carry out investigations of asymptomatic infections and epidemiological analysis and research; improve prevention and control measures; revise and improve prevention and control plans and diagnosis and treatment plans, and scientifically respond to the risk of infection caused by asymptomatic infections.

The second is to increase screening and monitoring. Intensify screening in a targeted manner, and expand the scope of testing to close contacts, key areas, and key populations of discovered cases and asymptomatic infected persons. Combining with the reality of resuming work and resuming production and school, strengthen the monitoring of key cities, key populations, and key places to find hidden dangers to the greatest extent. Take precautions against cross-border import and export of the epidemic, and conduct nucleic acid testing on all entry personnel. After discovering asymptomatic infections, timely conduct epidemiological investigations, find out the source and release information openly and transparently.

The third is to strengthen management and treatment. Once an asymptomatic infection is found, it is necessary to immediately follow the “four early” requirements, strictly centralized isolation and medical management, and conduct isolation medical observation for close contacts. Symptoms occurred during isolation and were immediately transferred to a designated medical institution for treatment.

The fourth is to strengthen group defense and group control. Adhere to the combination of group and professional expertise, increase the spread of epidemic prevention knowledge, guide the public in scientific protection, conduct extensive training, and improve the prevention and control capabilities and levels of grassroots disease control personnel, medical personnel, and community workers.

**Figure 1:**
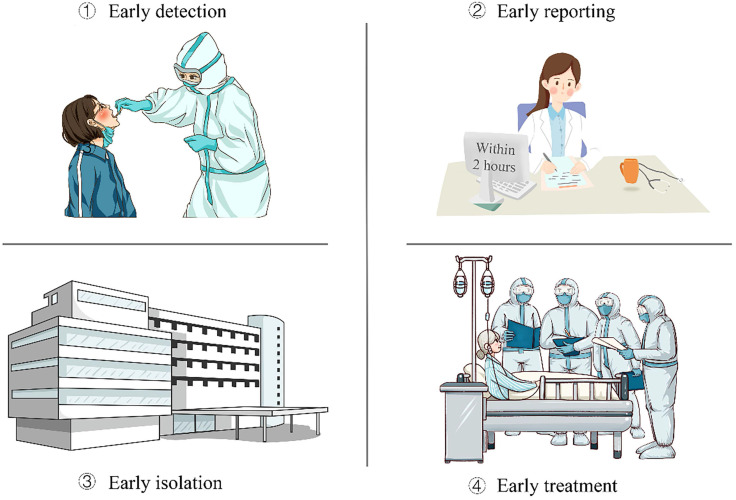
The strategy of the Chinese government in the management of asymptomatic patients with COVID-19.

The key to all of these policies is “four early” (**Figure 1**):

1) Strengthen the monitoring and early detection of asymptomatic infections. The Chinese government has proposed some specific measures: first, active detection of close contacts of new coronary pneumonia cases during medical observation; second, active detection during cluster epidemic investigation; third, in the process of tracing the source of infection of new coronary pneumonia cases active testing of exposed populations; fourth, active testing of some people living in areas where new coronary pneumonia cases continue to spread at home and abroad; fifth, relevant personnel found in epidemiological investigations and opportunistic screening.

2) Standardize the early reporting of asymptomatic infections. The government requires all types of medical and health institutions at all levels to report asymptomatic infections through the Internet within 2 hours. After receiving reports of asymptomatic infections, county-level disease control agencies complete case investigations within 24 hours, register close contacts promptly, and report case investigation forms or investigation reports through the infectious disease report information management system promptly. After the asymptomatic infection is released from the centralized medical observation, the medical and health institution shall fill in the date of the release of the medical observation in the infectious disease report information management system.

3) Strengthen the isolation management of asymptomatic infections. Asymptomatic infections were intensively medically observed for 14 days. Those with clinical symptoms and signs related to new coronary pneumonia were converted into confirmed cases during the period. Those who have been in intensive medical observation for 14 days and whose nucleic acid test is negative for two consecutive samples (at least 24 hours apart) are removed from intensive medical observation, and those who are still positive in nucleic acid test and have no clinical symptoms need to continue intensive medical observation. For asymptomatic infections who have been relieved of centralized medical observation, medical observation and follow-up will continue for 14 days. After the intensive medical observation was released, the patients should go to the designated hospital for follow-up visits in the second and fourth weeks to continuously monitor their health status. Close contacts of asymptomatic infected persons should be intensively medically observed for 14 days.

4) Treat as soon as possible. Asymptomatic infected persons have clinical manifestations during the intensive medical observation period. They should be immediately transferred to designated medical institutions for standardized treatment. The treatment of COVID-19 patients adopts the principles of pooling patients, experts, resources, and treatment.

## Conclusion

In conclusion, there is a certain proportion of asymptomatic COVID-19 infection in different countries and regions. Asymptomatic infection is contagious, and there is a risk of transmission. Therefore, identifying the asymptomatic infected persons and cutting off the infection source is an effective way to prevent the spread of this disease. Asymptomatic patients have hidden clinical symptoms. So, screening based only on the clinical symptoms of COVID-19 can easily lead to a missed diagnosis, forming loopholes in prevention and control measures. Therefore, determining asymptomatic infection patients by SARS-CoV-2 nucleic acid testing has very important epidemiological significance for controlling the source of infection, especially in the close contacts of COVID-19 patients. A series of prevention and control measures adopted by the Chinese government, especially the “Four Early” policy, have achieved outstanding achievements worth learning from by other countries.

## Figures and Tables

**Table 1:** The incidence of asymptomatic infections of SARS-CoV-2 in different studies

Country/region	Objective and method	Prevalence	Asymptomatic
Spain 10	35 883 households were selected from municipal rolls using two-stage random sampling stratified by province and municipality size, with all residents invited to participate.	5.0% (3053/61075)	21.9%-35.8%
The United States 11	Using random sampling with 16 025 people of all ages taken from 23 March to 12 May at 10 geographical sites in the US	1.0%-6.9%	40.0%
Long-term care facilities, Belgian 12	Using random sampling stratified with residents and staff invited to participate, giving priority to facilities with a higher number of suspected cases	3.0% (8343/280427)	74.8% (6244/8343)
Iceland 13	Testing to persons living in Iceland who were symptomatic, had recently traveled to high-risk countries, or had contact with infected persons. Using two strategies: issuing an open invitation to 10,797 persons and sending random invitations to 2283 persons	0.8% (100/13080)	43.0% (43/100)
China 14	All COVID-19 cases reported in mainland China infectious disease reporting information system as of 11 February 2020 were selected	N/A	1.2% (889/72314)
Ningbo, China 15	2147 close contacts of COVID-19 cases were selected from Ningbo using prospective research methods	6.2% (132/2147)	16.7% (22/132)
Taiwan, China 16	This prospective case-ascertained study in Taiwan included 2,761 close contacts of 100 confirmed COVID-19 patients	0.8% (22/2761)	18.2% (4/22)
Skilled Nursing Facility, Washington 4	Twenty-three days after the first positive test result in a resident at this skilled nursing facility, using random sampling with 89 residents and staff members were tested for SARS-CoV-2	64.0% (57/89)	5.3% (3/57)
Senior independent and assisted-living community, Washington 17	After two residents of a senior independent and assisted living community in Washington were hospitalized with confirmed COVID-19 infection, all residents and staff members were tested for SARS-CoV-2	4.2% (6/142)	N/A
Japan 18	A total of 565 Japanese citizens were evacuated from Wuhan, China on three chartered flights were tested for SARS-CoV-2	1.4% (8/565)	50.0% (4/8)
“Diamond Princess” cruise ship 19	The cruise ship hosting 3,711 people underwent a 2-week quarantine and were tested for SARS-CoV-2	17.1% (634/3711)	51.7% (328/634)
